# The Potential of *Hibiscus sabdariffa* Linn in Inducing Glucagon-Like Peptide-1 via SGLT-1 and GLPR in DM Rats

**DOI:** 10.1155/2019/8724824

**Published:** 2019-11-11

**Authors:** Neng Tine Kartinah, Fadilah Fadilah, Ermita Ilyas Ibrahim, Yuliana Suryati

**Affiliations:** ^1^Department of Medical Physiology, Faculty of Medicine Universitas Indonesia, Jakarta 10430, Indonesia; ^2^Department of Chemistry, Faculty of Medicine Universitas Indonesia, Jakarta 10430, Indonesia; ^3^Master Program in Biomedical Sciences, Faculty of Medicine Universitas Indonesia, Jakarta 10430, Indonesia; ^4^Faculty of Health and Agricultural Sciences, Indonesia Catholic University of Saint Paul Ruteng, Manggarai 86511, Indonesia

## Abstract

**Background:**

Glucagon-like peptide 1 (GLP-1) hormone is an incretin hormone that is secreted in the ileum and plays a role in the pancreas to increase insulin secretion, stimulate proliferation, and prevent pancreatic *β*-cell apoptosis. Currently, diabetes mellitus (DM) treatment based on GLP-1 work is being developed, for instance, from herbal plants such as *Hibiscus sabdariffa* Linn (*H. sabdariffa*). Therefore, this study aims to determine the potential of *H. sabdariffa* in GLP-1 secretion in the ileum and its action in pancreatic *β*-cells. In addition, this study also aims to determine the active ingredients of *H. sabdariffa* (Hib) that interact with sodium-glucose cotransporter-1 (SGLT-1) so that it can increase GLP-1 secretion in the ileum and interact with GLP-1 receptors (GLP-1R) in the pancreas.

**Method:**

This experimental study used 24 experimental animals of Sprague–Dawley type (aged 8–10 weeks, weight 200–250 g) that were divided into 6 groups, namely, (i) normal (C), (ii) normal-Hib 200 (C-Hib200), (iii) normal-Hib 500 (C-Hib500), (iv) DM (C-DM), (v) DM-Hib200, and (vi) DM-Hib500. *H. sabdariffa* extract was given orally once a day for 5 weeks. Testing of GLP-1 levels in the ileum and pancreatic tissue was performed by enzyme-linked immunosorbent assay. The prediction of the interaction mechanism of the active substance *H. sabdariffa* against GLP-1 was done using molecular docking.

**Results:**

There was a decrease in GLP-1 levels in the ileum of DM rats (*p* < 0.05). However, DM rats administered *H. sabdariffa* 500 mg/kg BW had GLP-1 levels that were the same as in normal rats (*p* > 0.05). This is due to active ingredients such as leucosin, which binds to SGLT-1. Administration of 500 mg/kg BW *H. sabdariffa* in DM rats resulted in GLP-1 levels in the pancreas that were the same as in normal rats (*p* > 0.05). In addition, the active ingredient of *H. sabdariffa*, delphinidin, binds to GLPR in the pancreas.

**Conclusion:**

The active ingredient of *H. sabdariffa* can increase GLP-1 secretion in the ileum and can interact with G protein-linked receptors in the pancreas.

## 1. Introduction

Diabetes mellitus (DM) is the third leading cause of death in the world because of the complications it causes [1]. In addition, the number of sufferers in the world continues to increase. In 2015, there were 415 million people with DM, and it is estimated that by 2040, there will be 642 million people with the disease [2]. Therefore, serious treatment efforts and proper handling of patients are needed so that DM does not cause complications that can result in death.

Currently, DM therapy includes antidiabetes drugs that have an effect on insulin secretion, but comprehensive treatment that is not only able to increase insulin secretion but also able to prevent damage to pancreatic *β*-cell and even stimulate the proliferation of new *β*-cells in the pancreas has yet to be developed. Glucagon-like peptide 1 (GLP-1) hormone is an incretin hormone that has the role of increasing insulin secretion as well as stimulating the proliferation and preventing apoptosis of pancreatic *β*-cells. Thus, GLP-1 provides a new hope in the treatment of DM [3, 4]. In addition, GLP-1 also plays a role in suppressing appetite and inhibiting gastric emptying. These effects provide benefits for handling DM as a whole [4].

Currently, mimetic/analog GLP-1 drugs that have an effect of working the GLP-1 hormone, such as liraglutide, exenatide, and drugs that inhibit the degradation of the hormone GLP-1 by the dipeptidyl peptidase-4 (DPP-4) enzyme such as sitagliptin, vildagliptin, and saxagliptin, have been developed [5, 6]. However, no drugs have yet been developed to improve their secretions. GLP-1 was secreted mainly from open-type enteroendocrine L cells in the ileum and colon. Release of GLP-1 was regulated by several factors, including nutrition such as glucose. Glucose is the most powerful stimulus for GLP-1 secretion. Glucose is detected by L-cells and then transported on the luminal surface of L-cells through the sodium-glucose cotransporter-1 (SGLT-1) so that it will increase GLP-1 secretion [7].

Secretion of GLP-1 has been proven to be influenced by herbal plants. Yu et al. [8] showed that Huang-Lian-Jie-Du-Decoction herbal administration for 5 weeks in DM rats increased proglucagon mRNA for synthesis of GLP-1 and secretion GLP-1 [9]. This is an opportunity to develop herbal-based treatments. The development of the use of herbal plants requires attention, because although herbs are beneficial for the treatment of DM, they are also associated with minimal side effects.

One of the herbs often used as an alternative treatment for DM is *Hibiscus sabdariffa* Linn (*H. sabdariffa*), or rosella (red tea) [10, 11]. However, the potential of *H. sabdariffa* in increasing GLP-1 secretion in the ileum was not known. *H. sabdariffa* has the potential as a treatment for DM because the flower part has an active substance that contains anthocyanins (flavonoid-type polyphenols) [11, 12]. At present, it is known that *H. sabdariffa* has the potential to inhibit the activity of the enzyme DPP-4. The enzyme plays a role in catalyzing and drastically reducing the biological activity of GLP-1, so that the half life of GLP-1 is not long, only about 8–10 minutes [13].

Another potential mechanism of *H. sabdariffa* that can be used in the management of DM is the increased production of insulin. The administration of 200 mg/kg body weight (BW) of *H. sabdariffa* in DM with streptozotocin induction (STZ) can increase insulin production [14, 15]. However, it is still unknown whether this increase in insulin production was related to the function of GLP-1. GLP-1 binds to the GLP-1 receptor (GLP-1R) in pancreatic *β*-cells and activates adenylate cyclase (AC), which causes the formation of cyclic monophosphate adenose (cAMP), which in turn stimulates insulin secretion in pancreatic *β*-cells [16].

Therefore, the purpose of this study was to determine the potential of *H. sabdariffa* in the secretion of GLP-1 in the ileum through its active compound bond with SGLT-1 and to determine the potential of *H. sabdariffa* in the pancreas through the bonds of its active compounds with GLP-1R.

## 2. Method

### 2.1. Study and Design

This study used 24 experimental male Sprague–Dawley rats (*Rattus norvegicus*) aged 8–10 weeks with a body weight between 200 and 250 g. This research received the approval of the FKUI Health Research Ethics Committee (No. 1172/UN2.F1/ETIK/2017). Before and during the treatment, the health of the rats was maintained to prevent illness. Experimental animals are given standard food and drink ad libitum. Lighting was set at a 12-hour light-dark cycle. The ambient temperature was maintained at 23 + 1°C.

### 2.2. Animals

Rats were divided randomly into 6 groups, namely, (i) normal rats (C), (ii) normal rats administered *H. sabdariffa* Linn 200 mg/kg BW/day (C-Hib200), (iii) normal rats administered *H. sabdariffa* Linn 500 mg/kg BW/day (C-Hib500), (iv) DM rats (C-DM), (v) DM rats administered H. sabdariffa Linn 200 mg/kg BW/day (DM-Hib200), and (vi) DM rats administered *H. sabdariffa* Linn 500 mg/kg BW/day (DM-Hib500).

The condition of the diabetic rats was induced by STZ injection (Nacalai, Japan) at a dose of 30 to 35 mg/kg BW in 0.1 M cold buffer citrate pH 4.5 intraperitoneally. After 72 hours of induction, blood was taken from the vein in order to measure blood glucose levels using an Accu-check glucometer. Rats were classified as diabetic if their blood glucose levels were >200 mg/dl. If the blood glucose level did not reach >200 mg/dl, the rats were again injected with STZ at the same dose. The group of normal (C) rats was injected with only buffer citrate (pH 4.5).

### 2.3. Provision of H. sabdariffa Methanol Extract

Simplicia of *H. sabdariffa* plants was obtained from the Center for Biopharmaceutical Studies at the Bogor Agricultural University. Extraction was carried out by maceration method using methanol, which was carried out at the Center for Biopharmaceutical Studies, Bogor Agricultural University. Dilution of the extract preparation was prepared for 7 days of treatment to avoid damage to the preparation. Then, the preparation was stored at 4°C.

The body weight of the rats was measured to determine the amount of *H. sabdariffa* extract to be given. Rats were administered extract doses of 200 mg/kg BW/day for the C-Hib 200 and DM-Hib 200 and 500 mg/kg BW/day for the C-Hib 500 and DM-Hib 500 groups. *H. sabdariffa* extract were administered orally using an injection syringe once a day for 5 weeks. In the normal and DM groups, only 2 mL of aquabides was given orally using a syringe once a day for 5 weeks.

### 2.4. Sampling Technique

The sample used consisted of ileum and pancreatic tissue. Before sampling at the end of treatment, rats were given orally a glucose load of 2.5 g/kg BW. The rats were then anesthetized completely through intraperitoneal injection using a combination of 0.01 ml/kg BW xylazine hydrochloride and 0.05 ml/kg BW ketamine. After 30 minutes of glucose loading, rats were decapitated, and segments were removed from the distal ileum (4 cm above the junction with the cecum) and pancreas. The dissected tissue was then cleaned and weighed, doused with 0.9% NaCl, and then stored at –80°C for GLP-1 analysis.

### 2.5. Molecular Docking Prediction

The molecular docking process begins with the formation of the desired protein for the screening process through nuclear magnetic resonance (NMR) spectroscopic technique, which is used to visualize the magnetic resonance of the core reacted with protein. The protein formation process uses a protein database along with a fairly complete protein substrate, namely, GDP-RCSB. Next, molecular docking was performed using software applications.

In silico screening used a journal database that aims to determine the interaction of active molecules that have activity on GLP-1 [17]. In silico work was done to filter approximately 16 compounds in *H. sabdariffa* by using the ligand base of the Autodock Vina device to molecular screening and binding. The active compounds in *H. sabdariffa* that are docked include chlorogenic acid, leucoside, 5-caffeoylquinic acid, quercetin, myricetin, tiliroside, caffeic acid, epigallocatechin, protocatechuic acid glucoside, delphinidin-3-sambubioside, cyanidin-3-sambubioside, 4-methylgallocatechin, 5-P-coumarylquine, kaempherol-3-glucoside, Α-coumarylquinic acid, and quercetin-3 glucoside.

The three-dimensional structure of the ligand (active compound from *H. sabdariffa*) used in this study was obtained from the Pubchem® database. The structure has been optimized in three dimensions using Chemdraw® V-2000. Protein crystallography was obtained from a protein bank's data (http://www.rcsb.org/pdb/results/) with SGLT1 (2XQ2) and GLP-1R (5VEW). The Chimera program was used for the preparation of crystallographic proteins.

Validation of crystallographic ligands was carried out through four phases. Crystalline ligands were chosen for docking. Ligands from the docking process were stored and compared with crystallographic ligands to measure the root mean square deviation (RMSD). Docking results with RMSD <2 were selected. Protein docking and ligand derivatives were included in the PyRx program. Docking results were saved in csv and sdf formats. Analysis was carried out based on the interaction between residues and observed ligands along with binding affinity of molecular docking.

### 2.6. Data Analysis

A normality test using the Shapiro–Wilk test was conducted on the collected data. If the data were normally distributed, parametric tests using a one-way analysis of variance (ANOVA) were conducted, and if the data were not normal and remained abnormal after transformation, a nonparametric test was carried out using the Kruskal–Wallis test. A *p* value <0.05 was considered statistically significant. Data processing was carried out using the SPSS 23 computer program (Statistical Social Sciences 23).

## 3. Results

### 3.1. The Effects of H. sabdariffa on Ileum GLP-1 Levels

**Figure 1:**
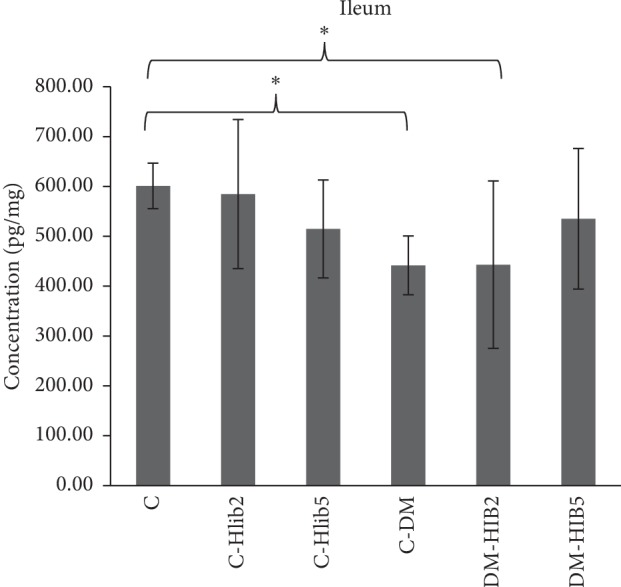
Average levels of GLP-1 in the ileum (pg/mL). C Normal control; C-Hib2: Hib-control200; C-Hib5: Hib500-control; C-DM: DM control; DM-Hib2: DM-Hib200; DM-Hib5: DM-Hib500. There were significant differences between group C with C-DM and DM-Hib2 (^*∗*^*p* < 0.05).

In the DM conditions, GLP-1 levels were lower than those in normal conditions, shown in Figure 1. GLP-1 levels in the normal group (C) were significantly higher than those in the DM group (*p* < 0.05) and the DM-Hib200 group (*p* < 0.05). GLP-1 level on diabetic rats given of *H. sabdariffa* at doses of 500 mg/kgBw/d (DM-HIB5) were on par from the normal control group (C). This indicated that the administration of *H. sabdariffa* Linn 500 mg/kg BW is able to maintain GLP-1 levels in the normal range (*p* > 0.05). The ability of *H. sabdariffa* Linn to maintain GLP-1 levels in DM conditions was proven by the docking results in Table 1.

### 3.2. The Effects of H. sabdariffa on Pancreas GLP-1 Levels

**Figure 2:**
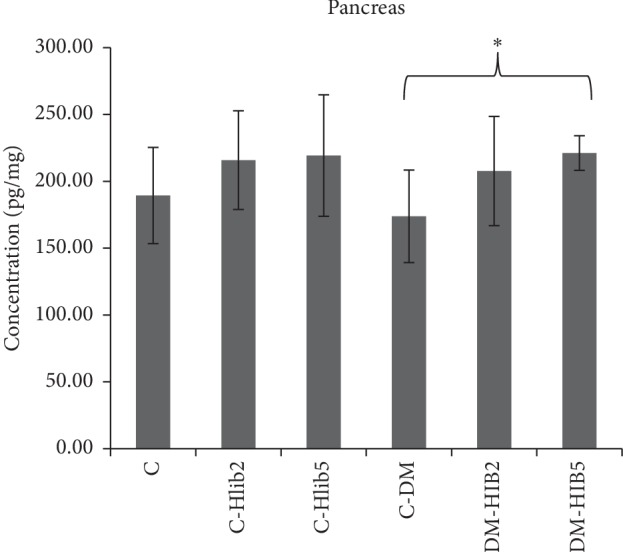
Average GLP-1 levels in the pancreas (pg/mL). C: normal control; C-Hib2: Hib-control200; C-Hib5: Hib500-control; C-DM: DM control; DM-Hib2: DM-Hib200; DM-Hib5: DM-Hib500. There was a significant difference between the C-DM group and DM-Hib2 group (^*∗*^*p* < 0.05).

The results of the examination (Figure 2) show that the DM group had the lowest levels of GLP-1 in pancreatic tissue. Administration of 200 mg/kg BW and 500 mg/kg BW of *H. sabdariffa* Linn in the DM group increased GLP-1 levels higher than that in the DM control group. The results of the one-way ANOVA followed by post hoc Tamhane test showed significant differences in GLP-1 levels between the DM groups with the administration of 500 mg/kg *H. sabdariffa* (DM-Hib500) and the DM control group (C-DM) (*p* < 0.05). Pancreas GLP-1 level on Diabetic rats given of *H. sabdariffa* at doses of 500 mg/kgBw/d (DM-HIB5) were on par from the normal control group (C). This indicated that the administration of *H. sabdariffa* Linn 500 mg/kg BW is able to maintain GLP-1 levels as normal (*p* > 0.05).

### 3.3. Molecular Docking Prediction between Active Compounds H. sabdariffa and SGLT1

**Figure 3:**
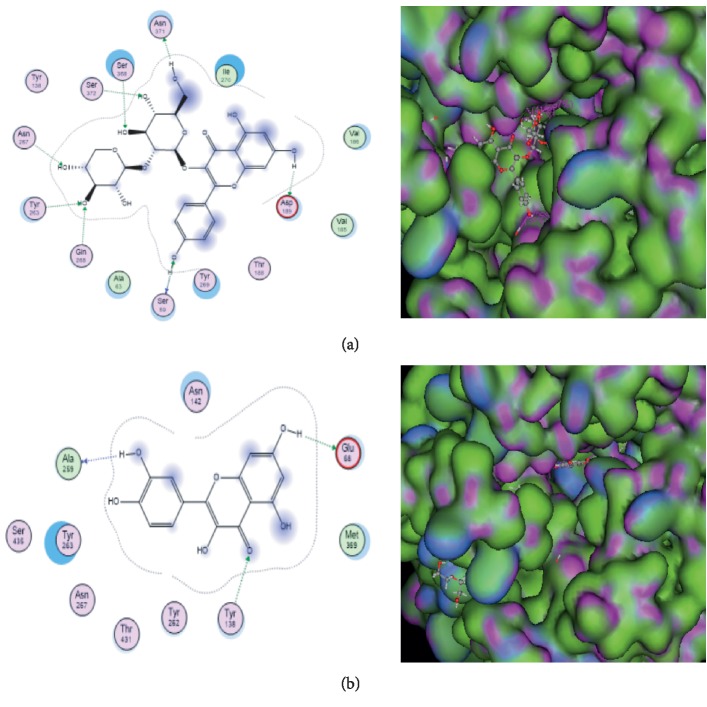
Molecular docking results of active compounds. (a) Leucosida with SGLT-1. (b) Quercetin with SGLT-1. The interaction occurs from the active side of the compound with the SGLT-1 receptor so that it can increase GLP-1 secretion.

Molecular docking shows the interaction between active compounds *H. sabdariffa* and glucose receptors in L ileum cells (SGLT-1) with a GDP code of 5VEW and has an activating function (as an activator). The active compounds that interact with SGLT-1 are leucoside, caffeic acid, protocatechuic acid glucoside, delphinidin-3-sambubioside, 5-P-coumarylquine, kaempherol-3-glucoside, and quercetin-3 glucoside. The complex between *H. sabdariffa* active compounds and SGLT-1 occurs in the active sites ser59, Lys285, and Val288. Leucoside and delphinidin bind to the binding sites of SGLT-1 receptors through residues Ser59. Caffeic acid and kaempherol-3-glucosides were bonded to the binding site of SGLT receptors through Lys 285 residues. Caffeic acid and quercetin-3 glucoside bind to receptor binding sites of SGLT-1 through Val288 residues (Table 1). The results of docking showed that leucoside and quercetin were bound to the SGLT1 receptor with the highest binding affinity compared with the others (Figure 3).

### 3.4. Prediction of Molecular Docking between Active Compounds H. sabdariffa and GLP-1R

**Figure 4:**
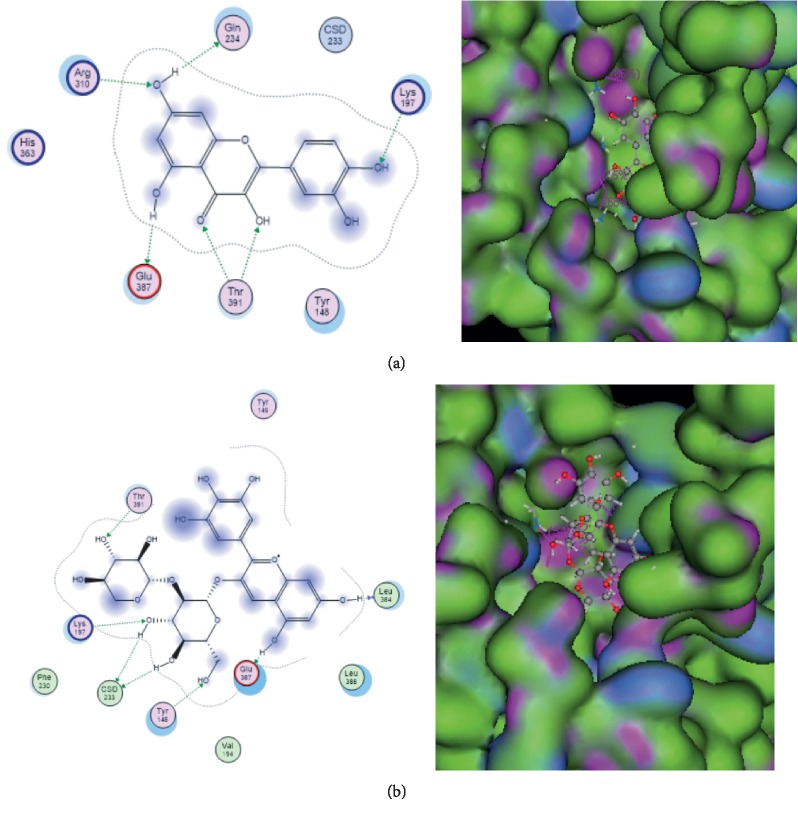
Results of molecular docking of active compounds. (a) Leucoside with GLP-1R. (b) Delphinidine with GLP-1R. There is an interaction of the active side of the compound with the GLP-1R so that it can increase GLP-1 levels and insulin secretion.

Molecular docking shows the interaction between the active compound of *H. sabdariffa* and the GLP-1R in pancreatic *β*-cells with the 3B4D GDP code on the active site modulator (Table 2). The results of docking showed that delphinidin and leucoside were bound to the GLP-1R receptor with the highest binding affinity compared with the others (Figure 4). Common residues found in interactions with GLP-1Rs are Tyr148, Tyr152, Tyr241, and Arg310. Leucoside, delphinidin, kaempherol-3-glucoside, and tiliroside bind to the binding site of the GLP-1R via residue Tyr148. Cyanidin-3-sambubioside, 5-P-coumarylquine, and protocatechuic acid glucoside bind to GLP-1R binding sites through the Tyr148 and Tyr152 residues, and caffeic acid and Α-coumarylquinic acid bind with the GLP-1R binding site through residues Tyr152 and Arg310.

## 4. Discussion

### 4.1. The Potential of Hibiscus sabdariffa Linn in SGLT-1

This study showed that DM conditions decreased GLP secretion [1]. According to Jung et al., the decrease in GLP-1 levels in DM patients was 53% when compared with the normal group [16]. Decreased GLP-1 secretion in type 2 DM were caused by damage to the proglucagon posttranslation process, because GLP-1 is a product of the proglucagon gene posttranslation process by converting prohormone. In addition, the decrease in GLP-1 secretion in DM was also caused by impaired GLP-1 response to oral nutrition in individuals with DM [18]. Oral nutrition, such as glucose, is one of the factors that influence the secretion of GLP-1. Glucose enters through SGLT-1 along with sodium, resulting in cell membrane depolarization. This opens the calcium channels and allows the entry of calcium into cells. Increased intracellular calcium levels trigger exocytosis of vesicles containing GLP-1 on the basolateral surface of L-cells, resulting in GLP-1 secretion [18, 19].

However, this study shows that the secretion of GLP-1 in the ileum did not decrease in DM rats given a dose of 500 mg/kg *H. sabdariffa*. Thus, *H. sabdariffa* has the potential to maintain GLP-1 secretion in the ileum in DM conditions. This is due to the content of polyphenols and anthocyanins in *H. sabdariffa*, which has an effect on the secretion of GLP-1. This is in line with research using polyphenol extract by Fujii et al., who found that administration of caffeine-free coffee polyphenol extract (CPE) increases the levels of GLP-1 in blood. The GLP-1 level in the control group was 1 *μ*M, and after being given CPE extract at a dose of 0.8 mM, the GLP-1 levels increased by 9 M [20]. Several other studies also showed that polyphenols derived from various plant extracts, such as potatoes, coffee, chocolate, and grape seeds, have been shown to increase the secretion of GLP-1 [13, 21].

In their study, Gonzalez-Abuin et al. used anthocyanin from grape seed extract (GSE). Their results showed that GLP-1 levels in the group given GSE were 4.7 pM higher than in the control group (3.5 pM) [22]. The research conducted by Kato et al. stated that the anthocyanin, delphinidin 3-rutinoside, significantly increased the secretion of GLP-1, which was thought to originate from an increase in cytosolic Ca^2+^ resulting in CaMKII activation [23]. This is in line with the research conducted by Tani et al., who stated that anthocyanin derived from purple berry extract increased the endogenous secretion GLP-1 [24]. These results show that polyphenols and anthocyanins have the potential to increase GLP-1 levels [22].

However, the content of polyphenols and anthocyanins is not yet known to play a role in increasing the secretion of GLP-1. Some of the active compounds of polyphenols include leucoside, caffeic acid, protocatechuic acid glucoside, 5-P-coumarylquine, kaempherol-3-glucoside, and quercetin-3 glucoside, while the content of anthocyanin active compounds includes delphinidin-3-sambubiosid [17]. The results of this study have shown the interaction of active compounds of polyphenols and anthocyanins with SGLT-1 in ileum L cell transporters through molecular docking. This study has proven that *H. sabdariffa* works to activate SGLT-1 receptors in ileal L cells, resulting in an increase in GLP-1 secretion. There are several active ingredients of *H. sabdariffa* that can interact with SGLT-1 in ileal L cells, such as leucoside, caffeic acid, protocatechuic acid glucoside, delphinidin-3-sambubioside, 5-P-coumarylquine, kaempherol-3-glucoside, quercetin-3, and glucoside. Leucoside has the highest binding affinity compared with other ligands. However, this study was not able to explain the mechanism of signal transduction from the activation of SGLT-1 receptors by *H. sabdariffa* active compounds to the production and secretion of GLP-1 in ileal L cells. Nagamine et al. used the active compound caffeoylquinic acid from sweet potato extract, which has an impact on increasing GLP-1 secretion in murine cells, [21] but when compared with this study, caffeoylquinic acid has no interaction between SGLT-1. Caffeoylquinic acid is another mechanism related to the role of the active compound in increasing GLP-1 secretion.

### 4.2. The Potential of *Hibiscus sabdariffa* Linn in GLP-1R

GLP-1 was secreted by intestinal L cells that then go to pancreatic *β*-cells to bind to the GLP-1R. The bond of GLP-1 with its receptors activates several signals that have an impact on insulin secretion, increase proliferation, and prevention of *β*-cell apoptosis. Increasing insulin secretion occurs through the activation of AC, which forms cAMP. Then, cAMP stimulates the activity of protein kinase A (PKA) and exchange protein activated 2 (Epac2) so that there is an increase in intracellular calcium and insulin exocytosis from *β* pancreatic cells [3, 16, 25]. In addition, GLP-1 activity through the activation of PKA can reduce pressure in the endoplasmic reticulum, which can maintain *β*-cell function through activation of mitogen-activated protein kinase to promote proliferation and inhibit *β*-cell apoptosis. GLP-1 also stimulates the epidermal growth factor receptor transactivation process, which activates phosphatidylinositol-3 kinase (PI3K). PI3K activates the insulin receptor and protein kinase C, substrate which causes gene transcription and ultimately stimulates *β*-cell mass expansion, increases proliferation, and prevents apoptosis. This shows that the GLP-1 bond with its receptors can increase insulin secretion through *β*-cell proliferation and prevention of *β*-cell apoptosis [16].

This study shows the potential of *H. sabdariffa* in increasing the amount of GLP-1 levels that bind to the receptors in the pancreas. This is evidenced by the level of GLP-1 in the pancreas in the DM group, and those who were given a dose of 500 mg/kg showed a higher body weight than in the DM group. This is in line with the results of Yu et al., who found that subjects who received Chinese herbal plants (Huang-Lian-Jie-Du) had elevated levels of GLP-1 in *β* pancreatic cells [8]. Increased GLP-1 in the pancreas was caused by the ability of *H. sabdariffa* to maintain GLP-1 levels in circulation so that it can reach its target in the pancreas. It is known that GLP-1 has a short half life of about 30 seconds in circulation. GLP-1 is degraded and inactivated by the DPP-4 enzyme [26]. However, as a DPP-4 inhibitor, *H. sabdariffa* has a potential impact on increasing the amount of GLP-1 active in the pancreas [13].

In addition, this study shows the potential of *H. sabdariffa*, which can interact with the GLP-1R in the pancreas. Some of the active compounds of *H. sabdariffa* include those that bind to the GLP-1R in pancreatic *β* cells, such as leucoside (kaempferol-3-O-sambubioside), myricetin, tiliroside, caffeic acid, methylepigallocatechin (flavonoid polyphenols), cyanidin-3-ambubioside, and delphinidin-3-sambubioside (anthocyanin-type polyphenols). Delphinidin-3-sambubioside has the highest binding affinity compared with the other ligands. The interactions between the active compound *H. sabdariffa* and GLP-1R cause activation of the GLP-1 receptor and start the signal transduction thereby increasing the production and secretion of GLP-1. However, further research needs to be done to explain how the GLP-1 signaling pathway activated by compounds is active for synthesis of insulin, *β*-cell proliferation, and apoptosis. Thus, the content of the active compound *H. sabdariffa* can also act as a GLP-1 analogue.

## 5. Conclusion

The administration of *H. sabdariffa* 500 mg/kg BW in DM rats is able to maintain GLP-1 levels in the ileum as normal because the content of active compounds such as leucoside can interact with SGLT-1. However, further research was needed to determine the mechanism of signal transduction from the activation of the SGLT-1 receptor by the active compound of *H. sabdariffa* to the production and secretion of GLP-1 in ileal L cells.

The administration of *H. sabdariffa* 500 mg/kg BW is able to maintain GLP-1 levels in the pancreas as normal. In addition, *H. sabdariffa* can also act as a GLP-1 analogue. Its active compounds, such as delphinidin-3-sambubioside, can interact with GPLR in the pancreas. However, further research was needed on the GLP-1–activated signaling pathways by assessing proteins involved in transduction signals for *β*-cell proliferation and prevention of *β*-cell apoptosis.

## Figures and Tables

**Table 1:** Interaction of active compounds *H. sabdariffa* with SGLT-1 in ileum L cells.

Protein	Compound	ΔG	pKi	Hdon	Hacc
SGLT1: Glucose receptors in ileal L cells	Leucoside (kaempferol-3-O-sambubioside) (flavonoid)	–15.2041	20.346	Ser59; Ser59; Asp189; Tyr263; Ser368; Asn371; Ser372;	Ser59; Tyr263; Asn267; Gln268; Ser368; Ser372
	Cyanidin-3-sambubioside	–12.1706	10.117	Asn371; Ser372;	Tyr263; Asn267
	Caffeic acid	–8.4396	7.579	Val288	Lys285; Ser523
	Epigallocatechin (flavonoid)	–7.2361	5.869	Ser368; Asn371; Ser372	Gln268; Ser368
	Delphinidin (antosianin)	–9.3861	6.267	Ser59; Asp189; Tyr263	Asn267; Gln268
	Α-coumarylquinic acid	–7.5621	5.213	Ser368; Asn371; Thr391	Ser368; Ser372
	4-metilgallocatechin	–8.7321	5.141	Asn371; Csd233; Leu384;	Gln268
	5-P-coumarylquine	–10.4872	6.139	Ser59; Csd233; Csd233	Ser59; Tyr263
	Kaempherol-3-glucoside	–12.4321	9.119	Val288; Asn371; Ser372	Asn267; Gln268
	Protocatechuic acid glucoside	–9.4764	5.929	Ser59; Asp189; Tyr263; Ser368	Ser59; Tyr263
	Quercetin-3 glucoside	–8.4872	5.739	Asn371; Thr391	Lys285; Ser523
	Chlorogenic acid	–9.7845	10.058	Tyr309	His231; Tyr309
	5-caffeoylquinic acid	–9.6979	12.102		Lys124; Lys127; Arg273; Arg273; Arg273; Lys383
	Quercetin	–13.5351	14.982	Glu68; Ala259	Tyr138
	Myricetin	–11.7756	12.820	Glu88; Asn142; Ser435	Asn64; Ser435
	Tyliroside	–13.4242	10.705	Ser79; Gly230; Gly230	His231; Asn240

ΔG, mean binding energy; pKi, binding affinity; H don, hydrogen donor; H acc, hydrogen acceptor. Red font: active site receptor.

**Table 2:** Interaction of active compounds of *H. sabdariffa* with GLP-1R pancreatic cells.

Protein	Compound	ΔG	pKi	Hdon	Hacc
GLP-1R: GLP-1 receptor in pancreatic *β*-cells	Leucoside (kaempferol-3-O-sambubioside)	–14.3186	11.855	Tyr148; Csd233; Glu387	Tyr148; Arg150; Lys197
	Myricetin	–13.6855	14.116	Tyr148; Tyr152; Csd233; Tyr241; Glu364	Tyr148; Tyr152; Tyr241; Arg310; Arg310; Arg310
	Tiliroside	–13.5443	13.082	Tyr148; Lys147; Csd233; Glu387	Tyr148
	Cyanidin-3-sambubioside	–12.1706	5.117	Tyr148; Tyr152	Tyr148; Tyr152; Lys197; Lys197; Lys197
	Caffeic acid	–9.7629	9.597	Tyr152; Tyr152; Thr391	Tyr152; Tyr152; Lys197; Thr391
	Epigallocatechin	–11.2461	10.869	Tyr152; Tyr152; Asp198; Thr391	Tyr152; Tyr152; Lys197; Thr391
	Delphinidin	–14.3861	11.267	Tyr148; Csd233; Csd233; Csd233; Leu384; Glu387; Thr391	Tyr148; Lys197; Thr391
	Α-coumarylquinic acid	–12.5621	6.213	Tyr152; Tyr152; Thr391	Tyr152; Lys197
	4-metilgallocatechin	–11.7321	5.341	Tyr152; Lys197; Csd233; Leu384;	Arg310; Arg310
	5-P-coumarylquine	–10.4872	5.139	Tyr148; Csd233; Csd233	Tyr152; Lys197;
	Kaempherol-3-glucoside	–12.4321	6.119	Tyr148; Csd233; Leu384; Glu387; Thr391	Tyr148; Arg150; Lys197
	Protocatechuic acid glucoside	–9.1506	3.217	Tyr148; Tyr152	Tyr148; Lys197; Lys197; Lys197
	Chlorogenic acid (flavonoid)	–12.4111	10.534		Tyr153; Arg190; Arg190; Lys197; Gln234
	5-caffeoylquinic acid (flavonoid)	–13.9335	9.028	Glu412	Arg348; Arg348; Arg1095; Arg1095; Trp1126
	Quercetin	–11.9839	8.822	Gln234; Glu387; Thr391	Lys197; Arg310; Thr391; Thr391

ΔG, mean binding energy; pKi, binding affinity; Hdon, hydrogen donor; Hacc, hydrogen acceptor. Red font: active site receptor.

## Data Availability

The raw (excel) data used to support the findings of this study are available from the corresponding author upon request.
